# CAR-T bridging to allo-HSCT as a treatment strategy for relapsed adult acute B-lymphoblastic leukemia: a case report

**DOI:** 10.1186/s12885-018-5037-7

**Published:** 2018-11-20

**Authors:** Shupeng Wen, Zhiyun Niu, Lina Xing, Ying Wang, Hang Li, Na Kuang, Jianmin Luo, Xuejun Zhang, Fuxu Wang

**Affiliations:** 1 Department of Hematology, The Second Hospital of Hebei Medical University, Hebei Key Laboratory of Hematology, Shijiazhuang, 050000 Hebei China; 2 Department of Histology and Embryology, Hebei Medical University, Shijiazhuang, 050017 Hebei China; 3 Hebei Senlangbio Technology Co., LTD, Shijiazhuang, 050000 Hebei China

**Keywords:** Acute lymphoblastic leukemia, CAR-T cells, Allogeneic hematopoietic stem cell transplantation

## Abstract

**Background:**

Adults with relapsed acute lymphoblastic leukemia (ALL) have a poor prognosis, especially in patients who relapsed within 6 months of complete remission 1 (CR1). Allogeneic hematopoietic stem cell transplantation (allo-HSCT) is the treatment of choice. However, this can only be considered after complete remission 2 (CR2) is achieved. Therefore, bridging treatment is urgently needed.

**Case presentation:**

In the present study, we report a relapsed adult B-cell ALL case that achieved CR2 after treatment with CD19-directed chimeric antigen receptor (CAR)-modified T cell (CAR-T) therapy. After subsequent allo-HSCT, the patient acquired 21 months of disease-free survival.

**Conclusion:**

The present results confirm that both CAR-T and allo-HSCT are effective for treating refractory or relapsed B-ALL. However, a novel sequential treatment strategy with these two therapeutic methods may achieve longer disease-free survival time.

## Background

Relapsed acute lymphoblastic leukemia (ALL) remains as a challenging disease with very poor prognosis, particularly in adult patients. After first complete remission (CR1), which is relatively easy to achieve, it becomes very difficult to achieve a second complete remission (CR2) when the patient relapses again. In adult patients, CR2 rates are lower than 50% [1]. On the other hand, curative approaches, such as allogeneic transplantation, are only considered for ALL patients who relapse after achieving CR2. Thus, a bridging treatment is urgently needed for relapsed patients before they can receive allogeneic transplantation.

Targeted immunotherapy has emerged as a potential option for bridging treatment. The most promising targeted immunotherapy is CD19-directed chimeric antigen receptor-modified T cell (CAR-T) therapy, which has received more and more attention in recent years. Among all different targets, CD19-directed CAR-T has been the best studied target [2]. Basically, immune tolerance has to be overcome to enable the clearance of CD19^+^ leukemic B cells in ALL. Thus, autologous T cells are engineered to express chimeric antigen receptors (CARs) targeting CD19, which are linked to the intracellular signaling domains of the T-cell receptor complex. The ligation of CD19 monoclonal antibody on CAR-T cells to CD19 on B-ALL cells would thereby activate T cells and stimulate a potent cytotoxic response. However, chemotherapy can only induce complete remission (CR) in 18–45% of relapsed patients, with a median overall survival (OS) of 3–9 months. CD19-directed CAR-T therapy can achieve a high level of CR, especially in children and young adults, although some cases still relapsed [3, 4]. Nevertheless, treatment with CAR-T to achieve CR2, followed by allogeneic hematopoietic stem cell transplantation (allo-HSCT), may be an ideal treatment strategy for relapsed/refractory ALL patients.

The present study reports a case of relapsed adult acute B lymphoblastic leukemia, which acquired 21 months of disease-free survival after treatment with CAR-T, followed by allo-HSCT.

## Case presentation

A 42-year-old woman, who was admitted to the Department of Hematology, The Second Hospital of Hebei Medical University (Shijiazhuang, China) on May 6, 2016, presented with a one-month history of paleness and fatigue.

**Fig. 1:**
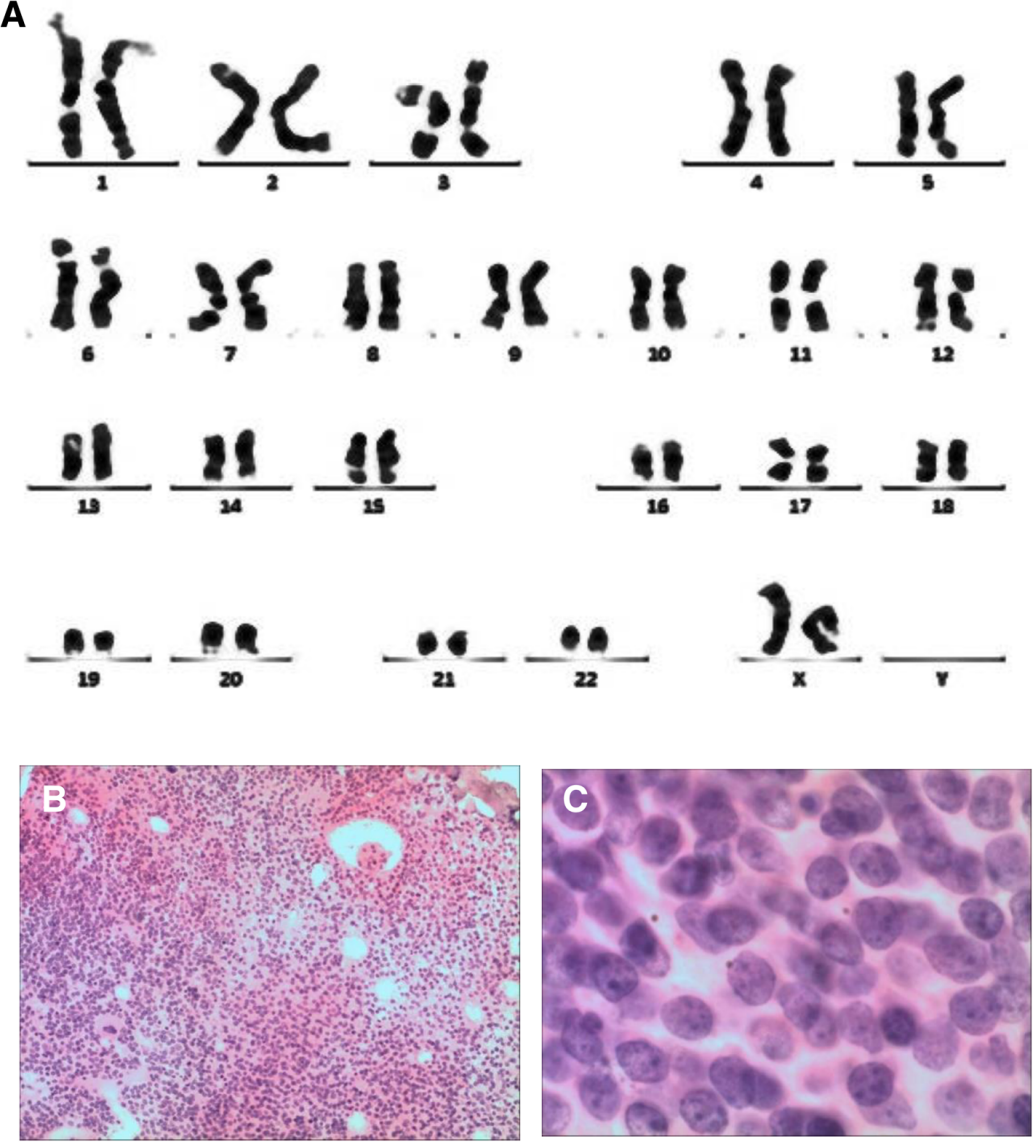
Cyto analysis of the patient. **a** The karyotype analysis of bone marrow cells using the R banding technique revealed a normal karyotype (46, XY) in the patient. **b** and **c** Histological analysis of bone marrow

**Fig. 2:**
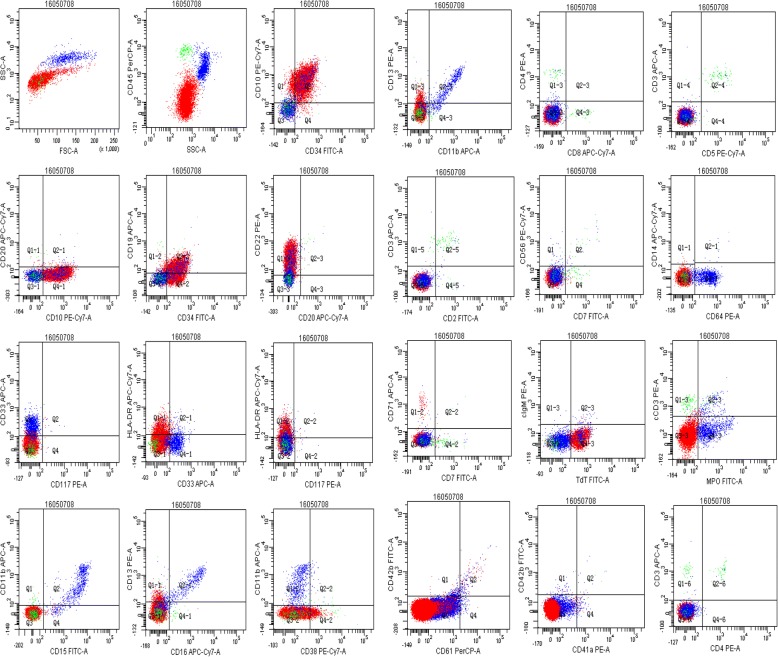
Immunophenotypic analysis by flow cytometry revealed a common B-cell acute lymphoblastic leukemia

The patient was a farmer with a history of tuberculous pleurisy 24 years ago. The hematological analysis revealed the following: white blood cell count (WBC), 23.8 × 10^9^/L; hemoglobin (Hb), 64 g/L; platelet count (PLT), 433 × 10^9^/L. Bone marrow and peripheral blood smears identified the proliferation of lymphoblastic cells (87% of bone marrow nucleated cells). Karyotype analysis revealed a normal karyotype (46, XX) [20] (Fig. 1). Immunophenotypic analysis by flow cytometry (FCM) revealed that blast cells accounted for 76.8%, which were positive for CD34, CD10, CD19, CD22 and HLA-DR, and negative for cIgM (Fig. 2). Hence, the diagnosis of common B-cell acute lymphoblastic leukemia was confirmed.

The patient received a standard induction chemotherapy regimen with dexamethasone, vincristine, daunorubicin, cyclophosphamide and peg-L-asparaginase, and subsequently achieved partial remission. Merely 5% of lymphoblasts were observed in the bone marrow smear. After receiving the second induction chemotherapy with vincristine, cyclophosphamide, mitoxantrone, cytarabine and dexamethasone, the patient was discharged from the hospital (June 18, 2016).

On July 22, 2016, the patient was admitted to our hospital again for consolidation. The complete blood count revealed the following: WBC, 4.1 × 10^9^/L; Hb, 82 g/L; PLT, 206 × 10^9^/L. The bone marrow examination revealed 41% of lymphoblasts. Therefore, early relapse was diagnosed. After one cycle of CAM (CTX, Ara-c and 6-MP) and two cycles of MA (methotrexate and cytarabine), 26% of lymphoblasts was found in the bone marrow. The leukemia in the patient was relapsed and refractory. Therefore, CAR-T therapy was introduced to the patient.

**Fig. 3:**
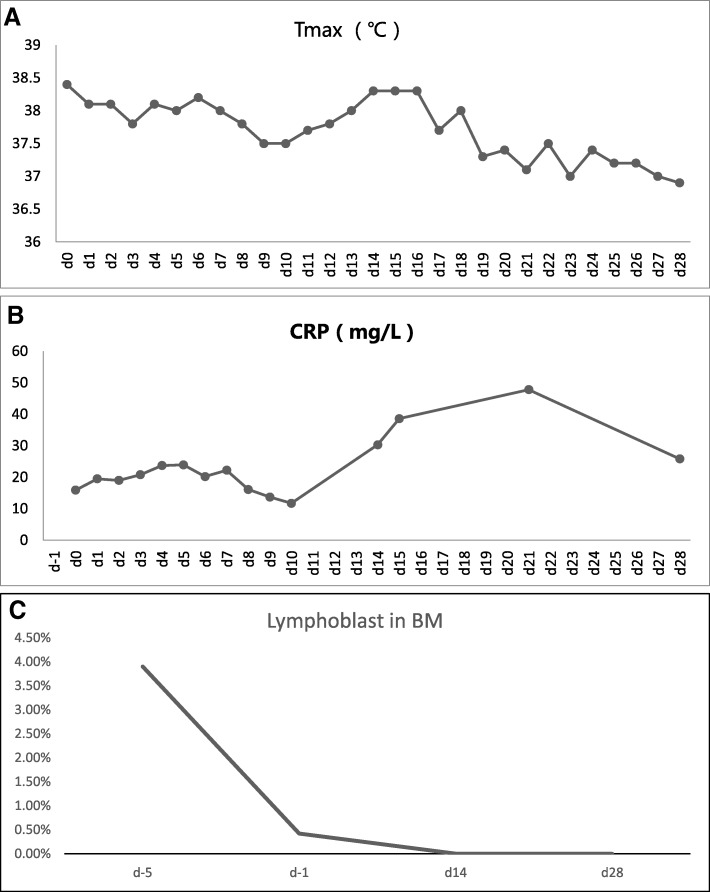
Changes within 28 days following CAR-T cell infusion in the body: **a** Body temperature; **b** C-reactive protein levels; **c** Number of lymphoblasts in the bone marrow

**Fig. 4:**
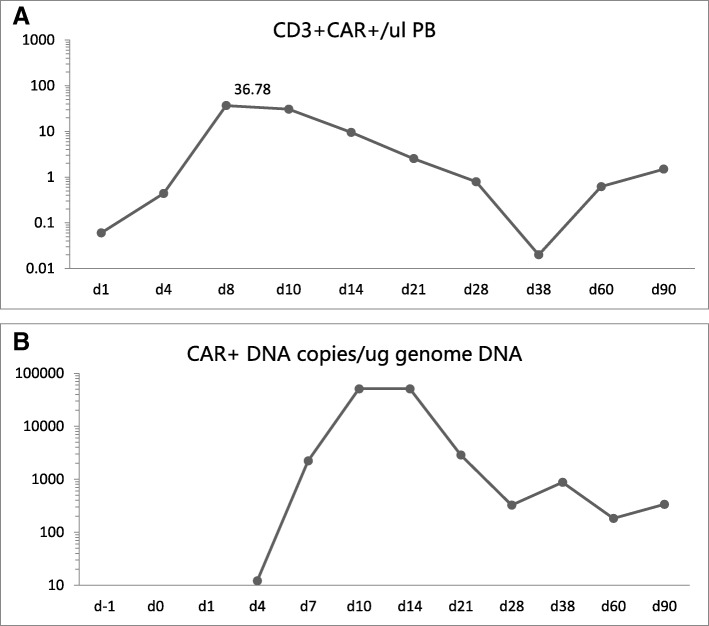
Changes within 90 days following CAR-T cell infusion. **a** The percentage of CD3^+^CAR^+^ T cells in total leukocytes, as analyzed by flow cytometery; **b** The copies of CAR DNA in peripheral blood detected by PCR

After the patient provided consent, 100 mL of peripheral blood was collected to prepare the anti-CD19 CAR-T cells. The CAR-T cells were engineered by Hebei Senlangbio Technology Co., Ltd. (Shijiazhuang, China). The construct of CD19-CAR comprised of the following: anti-CD19 scFv (FMC63), the CD28 transmembrane domain, 4-1BB costimulatory, CD3zeta activation domains, T2A autocleavage sequences, and endodomain-truncated EGFR. Three lentivirus package systems were used (psPAX2, pMD2.G and pLenti-EF1-CAR19), and these were co-transfected with JetPRIME (Polyplus Transfection) in 293FT cells. The lymphodepleting chemotherapy with the FC regimen (30 mg/m^2^ of fludarabine, day − 4 to − 2; 30 mg/kg of cyclophosphamide, day − 3 to − 1) started on November 5, 2016 (day − 4). On day zero, the patient received an infusion of anti-CD19 CAR-T cells, which were transfected by the Senl-B19 lentiviral vector to express the anti-CD19 CARs, and expanded with IL-2 and IL-7. The total dose was 5.0 × 10^5^ CAR-positive T-cells/Kg. Body temperature, C-reactive protein, CAR-T cell number in peripheral blood, the copy of CAR DNA, and leukemia cell level in the bone marrow were detected by FCM, as presented in Figs. 3 and 4.

On the day of reinfusion of CAR-T cells, the patient developed fever with a temperature of 38.4 °C. The temperature was lowered by NSAIDs and returned to normal after a week. The fever regenerated on the 14th day after transfusion, and body temperature returned to normal after 3 days of piperacillin treatment. On December 2, 2016, the blood examination revealed the following: WBC, 3.2 × 10^9^/L; Hb, 80 g/L; PLT, 354 × 10^9^/L. On December 6, 2016 (day 28), no lymphoblast was found in the bone marrow smear. The minimal residual leukemia examined by FCM was also negative, since no CD19^+^CD34^+^ lymphocyte was found in the bone marrow.

The patient has a human leukocyte antigen (HLA)-full matched brother, who was willing to be a donor for the hematopoietic stem cell transplantation (HSCT). On December 11, 2016, the patient underwent allogeneic transplantation with granulocyte colony-stimulating factor mobilized bone marrow cells plus peripheral blood hematopoietic stem cells from the patient’s brother. The preconditioning regimen was myeloablative, including cytosine arabinoside (2 g/m^2^) on day − 9, busulfan (3.2 mg/kg/day) on day − 8, − 7 and − 6, cyclophosphamide (1.8 g/m^2^/day) on day − 5 and − 4, and semustine (250 mg/m^2^) on day − 3. The graft-versus-host disease (GVHD) prophylaxis was based on cyclosporine, mycophenolate mofetil (MMF), and a short course of methotrexate. Donor mononuclear cells of 8 × 10^8^/Kg, including CD34 positive cells of 2.88 × 10^6^/Kg, were transfused. Neutrophil and platelet engraftments were observed on day + 10, while a full-donor-chimerism of 97.5% was evidenced on day + 28.

On day + 28, the patient had a seizure, accompanied by thrombocytopenia. Then, red-cell fragments were found in the peripheral blood smear. Other important abnormal results included elevated levels of serum lactate dehydrogenase (LDH), positivity for proteinuria (240 mg/dL), and a negative Coombs test. All these led to the diagnosis of transplantation-associated thrombotic microangiopathy (TA-TMA). Cyclosporin A was discontinued, a high dose of methylprednisolone (10 mg/kg) was used and tapered, and therapeutic plasma exchange (TPE) was performed daily for the first week, and on every second day for the second week, followed by plasma infusion every 3 days for 2 weeks. On August 4, 2017, no sign of seizure was present, blood examination revealed a WBC of 9.9 × 10^9^/L, a Hb of 77 g/L and a PLT 20 × 10^9^/L. Furthermore, the bone marrow smear revealed no lymphoblasts, and the minimal residual disease (MRD) assayed by FCM was negative. The DNA chimerism revealed a full-donor type, and the patient was discharged from the hospital.

## Discussion and conclusion

The present study reports an early relapse adult B-ALL case successfully treated by CAR-T therapy and allo-HSCT. Standard chemotherapy failed to achieve remission even after three cycles. Therefore, CD19-directed CAR-T therapy was attempted. The patient achieved CR2, which enabled her to be subsequently treated with allo-HSCT. The patient had no signs of leukemia recurrence or GVHD, and survived for more than 21 months.

Early relapse indicates the profound resistance of leukemic blasts to standard chemotherapy in B-ALL. For the 1,706 relapsed adult ALL patients, the overall CR rate after the first salvage was 40%. However, this rate decreased to 21% after the second salvage, and 11% after the third or greater salvage. The three-year survival rates in the first, second, and third or greater salvage were 11, 5 and 4%, respectively [5].

Although allo-HSCT remains the best therapeutic option for relapsed ALL, the effect widely varies in different disease states. The 5-year OS was 33% in CR2, and for those who could not achieve CR, the 5-year OS of a salvage transplant was only 8% [6]. It has been generally agreed that HSCT after achievement of the second CR is the ideal treatment of choice for relapsed ALL patients [7]. Therefore, it is very important to achieve the second CR.

A new cellular immunotherapeutic approach for relapsed ALL is the genetic modification of T cells to express a CAR specific for CD19. Several clinical trials of CD19-specific CAR-T cell therapy among children and adults with relapsed B-ALL have shown higher CR rates (70–90%), when compared to standard chemotherapy [8].

CAR-T cell therapies have shown promising results in relapsed and refractory ALL patients. Furthermore, the tolerability and response rates appear to be better in patients who have a lower leukemia load. The most commonly observed toxicity is cytokine-release syndrome (CRS). The second most common adverse event is CAR-T-cell-related encephalopathy syndrome (CRES), which can occur concurrently with or after CRS [9]. Park et al. reported side effects on 53 adults who received 19–28 z of CAR-T cells manufactured at MSKCC: severe CRS occurred in 14 of 53 patients (26%), and one patient died [3]. The persistence of CAR-T cells appears to be important to its efficacy [4].

There is still a need to evaluate the long-term outcomes of CAR-T therapies. Based on the limited data, CAR-T therapy alone may not be enough to achieve a high 5-year OS. In the report of Park et al., CR was observed in 83% of 53 patients. At a median follow-up of 29 months, the median event-free survival was 6.1 months, and the median OS was 12.9 months [3].

For patients who achieved CR after CAR-T therapy, the choice of treatment is very limited. HSCT may be a reasonable option. However, there is limited data to evaluate this strategy, and the results remain controversial. According to Park et al., among the 32 patients who had a MRD–negative CR, no significant difference was found in event-free survival and OS between patients who underwent transplantation and those who did not [3]. However, Pan et al. reported on 42 primary refractory/hematologic relapses, and nine refractory MRDs were detected by FCM. B-ALL patients were treated with optimized second generation CD19-directed CAR-T cells. Among these patients, 36 of 40 (90%) refractory/relapsed patients achieved CR or CR with incomplete count recovery (CRi), 23 of 27 CR/CRi patients received subsequent allo-HSCT and remained in MRD negative with a median follow-up of 206 days (45–427 days), and nine of 18 CR/CRi patients without allo-HSCT relapsed. These results indicate that CAR-T cells are effective for treating refractory or relapsed B-ALL, and subsequent allo-HSCT could further reduce the relapse rate [10].

The present case described a clinical application of CAR-T cell therapy, followed by allo-HSCT, for a refractory/relapsed B-ALL patient. Although the outcome is encouraging, more clinical trials are needed to better understand the benefits and limitations of this treatment strategy.

## Abbreviations

ALL: Acute lymphoblastic leukemia
allo-HSCT: Allogeneic hematopoietic stem cell transplantation
CARs: Chimeric antigen receptors
CAR-T cells: Chimeric antigen receptor T cells
CR1: Complete remission 1
CR2: Complete remission 2
CRES: CAR-T-cell-related encephalopathy syndrome
CRS: Cytokine-release syndrome
FCM: Flow cytometry
GVHD: Graft-versus-host disease
HLA: Human leukocyte antigen
MRD: Minimal residual disease
OS: Overall survival
TA-TMA: Transplantation-associated thrombotic microangiopathy
TPE: Therapeutic plasma exchange
